# Solid Phase Adsorption Toxin Tracking (SPATT) Technology for the Monitoring of Aquatic Toxins: A Review

**DOI:** 10.3390/toxins10040167

**Published:** 2018-04-20

**Authors:** Mélanie Roué, Hélène Taiana Darius, Mireille Chinain

**Affiliations:** 1 Institut de Recherche pour le Développement (IRD), UMR 241 EIO, P.O. box 53267, 98716 Pirae, Tahiti, French Polynesia; 2 Laboratory of Toxic Microalgae, Institut Louis Malardé (ILM), UMR 241 EIO, P.O. box 30, 98713 Papeete, Tahiti, French Polynesia; tdarius@ilm.pf (H.T.D.); mchinain@ilm.pf (M.C.)

**Keywords:** SPATT technology, passive monitoring, risk assessment, harmful algal blooms, aquatic toxins, microalgae, cyanobacteria

## Abstract

The Solid Phase Adsorption Toxin Tracking (SPATT) technology, first introduced in 2004, uses porous synthetic resins capable of passively adsorbing toxins produced by harmful microalgae or cyanobacteria and dissolved in the water. This method allows for the detection of toxic compounds directly in the water column and offers numerous advantages over current monitoring techniques (e.g., shellfish or fish testing and microalgae/cyanobacteria cell detection), despite some limitations. Numerous laboratory and field studies, testing different adsorbent substrates of which Diaion^®^ HP20 resin appears to be the most versatile substrate, have been carried out worldwide to assess the applicability of these passive monitoring devices to the detection of toxins produced by a variety of marine and freshwater microorganisms. SPATT technology has been shown to provide reliable, sensitive and time-integrated sampling of various aquatic toxins, and also has the potential to provide an early warning system for both the occurrence of toxic microalgae or cyanobacteria and bioaccumulation of toxins in foodstuffs. This review describes the wide range of lipophilic and hydrophilic toxins associated with toxin-producing harmful algal blooms (HABs) that are successfully detected by SPATT devices. Implications in terms of monitoring of emerging toxic risks and reinforcement of current risk assessment programs are also discussed.

## 1. Introduction

In aquatic (marine and freshwater) environments, some primary producers, in particular microalgae and cyanobacteria, are able to synthesize biotoxins that can be harmful to other organisms, including humans. Of the many thousands of microalgal species described, about 300 are involved in harmful events and more than 100 of these species produce potent and persistent natural toxins that can be harmful or even lethal to humans and animals [1]. Regarding cyanobacteria, around 100 species associated with the production of cyanotoxins are reported [2]. In freshwater environments, cyanobacteria (prokaryotes) are the main toxic microorganisms [3,4] whereas in marine environments, microalgae (eukaryotes) are primarily involved in toxic proliferation events also known as Harmful Algal Blooms (HABs) [4,5]. Recently, it has been shown that marine cyanobacteria can also produce toxins [6,7]. In this review, the term “HABs” will be used to refer to both toxin-producing microalgal and cyanobacterial blooms. HABs episodes represent a natural freshwater and marine risk for food safety and can have a significant health impact on fauna [8,9] as well as on human populations, even sometimes at a relatively low biomass in the case of species producing very potent toxins. Indeed, humans can be exposed to toxins through three major routes: (i) inhalation of toxic aerosols or direct contact with contaminated water/toxic microorganisms during bathing and recreational activities, causing respiratory issues and skin irritations [10,11], (ii) ingestion of contaminated drinking water [12], and (iii) ingestion of shellfish or fish that have accumulated toxins [13,14]. For instance, cyanobacteria are known to produce diverse cyanotoxins such as hepatotoxins, dermatotoxins, cytotoxins and neurotoxins [15]. Microalgae (mostly dinoflagellates but also diatoms) are involved in at least six well-described human poisoning syndromes caused by the consumption of toxic seafood: diarrheic shellfish poisoning (DSP), azaspiracid shellfish poisoning (AZP), ciguatera fish poisoning (CFP), neurotoxic shellfish poisoning (NSP), amnesic shellfish poisoning (ASP) and paralytic shellfish poisoning (PSP) [16].

Over the past decades, the occurrence of HABs have significantly increased globally, with for example the large worldwide increase of microcystin [17] or the emergence of tropical ciguatoxins in temperate waters of Europe [18], as well as their associated socioeconomic impacts (health, tourism, fishery, monitoring and management) [1,19]. However, it should be noted that this apparent important increase may be intensified by an increased interest in biotoxins and thus an increased monitoring of HABs. Although HABs’ response to global change needs to be considered with species-specific models [20], it is most likely that both marine and freshwater toxic HABs will be affected by climate change effects (e.g., ocean warming), as well as by increased anthropogenic pressures exerted on the aquatic environments globally (e.g., eutrophication). These observations have led to increased scientific awareness and regulatory policies in order to control/mitigate HABs and associated public health issues, e.g., through the development and implementation of novel technologies and approaches in research, monitoring and management programs.

Until recently, surveillance programs mostly relied on the survey of cell density of potentially toxic species and/or the monitoring of toxins in material intended for human consumption [5]. However, such methods have several limitations. For instance, cell-based monitoring programs are often limited to species known to be linked to particular toxin families, and allow only a snapshot of the composition of HABs populations at one time and place [21]. They are often laborious and time-consuming, and poorly adapted to the identification of small toxin-producing organisms (e.g., *Vulcanodinium rugosum* or *Azadinium spinosum*) or those with benthic/epiphytic life stages (e.g., *Gambierdiscus*, *Ostreopsis* or *Prorocentrum*). Moreover, identification of cryptic species through light microscopy is often inconclusive and requires the use of molecular analyses. Another significant issue inherent to toxin content variations in HABs species observed both at intra- and inter-species levels is that cell abundance for a given species cannot readily serve as a proxy of toxin concentrations in situ without the risk of potential false alarm [22]. As for shellfish and fish testing, this approach is also expensive, time-consuming, labor-intensive, and potentially subject to analytical interferences due to matrix effects issues. Furthermore, species-specific differences in toxin uptake and depuration as well as bio-transformation process of algal or cyanobacterial toxins in fish or shellfish increase the complexity and diversity of toxin profiles, making toxin identification and quantification even more challenging [23]. Supplementary toxin-based methods allowing the spatiotemporally integrated sampling of toxins directly in aquatic environments are thus needed for a more reliable assessment and management of the risks associated with HABs proliferation and related toxins.

This latter requirement has prompted the development of alternative cost-effective techniques such as the SPATT (Solid Phase Adsorption Toxin Tracking) method. This technology, first developed in 2004 by MacKenzie et al. [21] to monitor the occurrence of toxic algal blooms and shellfish contamination events in several locations of New Zealand, uses porous synthetic resins capable of adsorbing toxins directly from the water column. This methodology that allows the detection of dissolved toxic compounds directly in the environment, has several advantages [24]. In addition to low cost and simplicity of preparation, deployment, storage and transport, resins used to fill SPATT devices are relatively clean matrices thus simplifying and accelerating the extraction and analysis of toxins accumulated using this technology (e.g., fewer matrix effects, fewer compounds in extracts, lower detection limits). Furthermore, the profiles captured rather represent the metabolite profiles of microorganisms in the water column and can thus facilitate the tracking of toxin-producing microalgae. Finally, SPATT technology allows spatiotemporally integrated sampling of aquatic toxins and could thus provide unique information on toxin dynamics, such as the origin of new toxins, environmental persistence, and variations in the specific toxicity of producers [24]. Conversely, this recent technology also presents some limitations, such as a lack of calibration (no optimal deployment time, no validated sampling units, difficulties in translating laboratory data to field deployments) that is probably the main drawback preventing a more widespread adoption of this technology for monitoring and management [25]. Another major disadvantage is that SPATT devices allow the monitoring of dissolved toxins only. Indeed, in the case of toxins that bioaccumulate in foodstuffs, this raises the question of the real risk measured by this technology since dissolved toxins are not usually considered to be bioavailable for shellfish or fish as the currently accepted assumption is that the main mode for uptake of aquatic toxins is through consumption of particulate forms (i.e., through a digestive uptake) [26]. Nevertheless, some recent studies actually showed the substantial uptake of dissolved toxins in fish or shellfish, even though the mechanism of bioconcentration is still poorly understood [26]. Some authors have suggested an uptake of toxins from the dissolved phase through respiratory activities (i.e., through the gills) [27,28,29], or through drinking water (i.e., through gastrointestinal tract of fish) [28].

**Figure 1:**
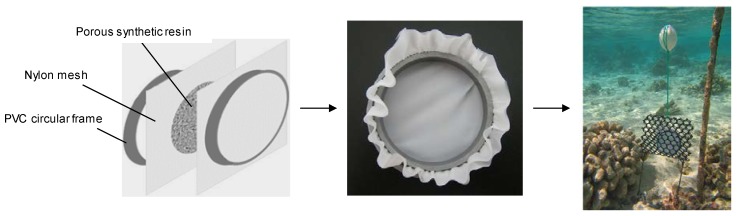
Example of one design used for the deployment of SPATT device in the field. Left: mode of assembly of SPATT device; Middle: SPATT device; Right: SPATT device deployed in the field. SPATT unit is made of two layers of nylon mesh filled with a porous synthetic resin, and fixed between two PVC circular frames. The device is then inserted in plastic grids to prevent its damage and excessive grazing by fish, and maintained in a vertical position in the water column using weights and floats. Reproduced with permission from Reference [32], Copyright Elsevier 2018.

Since their first introduction in 2004, the use of SPATT devices has continuously increased, from 19 peer-reviewed citations in 2004–2007 to 62 in 2008–2011 and 123 in 2012–2016 [25], and currently continues its rapid expansion. SPATT devices are conceptually similar to semipermeable membrane device (SPMD) or polar organic chemical integrative samplers (POCIS) that have already been used for other trace contaminants in water. They consist of a porous synthetic resin encased within an inert mesh, typically nylon, which is deployed in the laboratory or environment submerged in water (Figure 1). Several designs have been used for the construction of SPATT devices, e.g., PVC frames (Figure 1) [30,31,32], sewn bags [21,33,34], embroidery discs [23,33,34,35,36,37,38,39,40,41,42], Velcro mesh bags [43,44] or heat-sealed bags [45,46,47]. SPATT devices are then typically deployed in the field by attaching them to a structure or weighted line, and are often inserted in a “protection cage” (Figure 1) to prevent damages, especially in energetic environments, and grazing by fish.

Various adsorbent substrates with different chemical properties have been tested in order to detect a wide range of lipophilic and hydrophilic aquatic toxins. Aromatic type adsorbents based on a cross-linked polystyrenic matrix are by far the most used for the detection of lipophilic toxins, and some of them have also proven to be effective for the monitoring of hydrophilic toxins. However, numerous more adapted adsorbent substrates have also been tested for the detection of strongly polar hydrophilic toxins. Among all the resins tested, the Diaion^®^ HP20 appeared the most versatile and is by far the most commonly used adsorbent substrate (see Section 2 for more details).

Since its introduction more than a decade ago, the numerous laboratory and field studies conducted to assess the efficacy of SPATT technology have led to the conclusion that these passive samplers offer the ability to detect a wide range of aquatic toxins, in particular when combined with untargeted analysis using liquid chromatography coupled to high resolution mass spectrometry (LC-HRMS) [48]. This review presents an overview of the use of SPATT technology for the monitoring of some of the major lipophilic and hydrophilic toxins produced by microalgae and cyanobacteria in marine and freshwater environments. The implications in terms of monitoring of emerging toxic risks and reinforcement of risk assessment programs are also discussed.

## 2. Application of SPATT Technology for the Monitoring of Aquatic Toxins

### 2.1. Lipophilic Toxins

Lipophilic toxins (LTs) are produced by marine dinoflagellate microalgae. They are mostly harmful to humans through their bioaccumulation in foodstuffs (mostly shellfish, i.e., filter-feeding bivalves such as mussels, oysters, scallops and clams, but also fish), and can also cause damage to wildlife. Marine LTs are widely distributed in the world and some countries have established toxin regulations to protect public health or to secure exports. The application of passive SPATT technology for the monitoring of some LTs, i.e., diarrheic shellfish poisoning (DSP) toxins, yessotoxins (YTXs), azaspiracids (AZAs), cyclic imines (CIs) and ciguatoxins (CTXs), has thus been widely explored, by testing different adsorbent substrates (Table 1). The main in vitro and field studies conducted are reviewed and detailed below.

#### 2.1.1. Diarrheic Shellfish Poisoning (DSP) Toxins and Pectenotoxins (PTXs)

DSP toxins are produced by planktonic dinoflagellate species of the genus Dinophysis and benthic species of the genus Prorocentrum [60,61] but only the former produce pectenotoxins (PTXs) [62].

**Figure 2:**
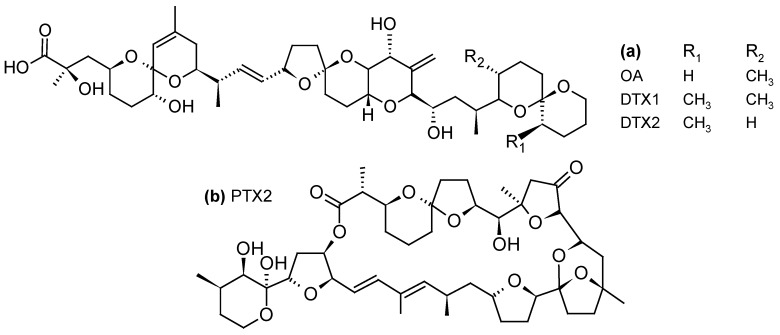
Structures of (**a**) okadaic acid (OA), dinophysistoxins 1 and 2 (DTX1, DTX2), and (**b**) pectenetoxin 2 (PTX2).

DSP toxins are acidic toxins and include okadaic acid (OA) and its derivatives known as dinophysistoxins (DTXs) (Figure 2a). These compounds are potent phosphatase inhibitors and lead to hyperphosphorylation of proteins involved in the cytoskeletal junctions that regulate the permeability of the cell, resulting in a loss of cellular fluids [63]. The main symptoms caused by the consumption of contaminated shellfish include gastrointestinal disorders such as diarrhea, nausea, vomiting, and abdominal pain [64].

PTXs are neutral toxins consisting of polyether-lactones (Figure 2b). Although no human intoxications by PTXs have been documented so far, liver damage such as the generation of vacuoles and deformation of hepatocytes have been observed following intraperitoneal (i.p.) injection of PTX2 in mice [65]. Oral administration of PTX2 also resulted in histopathological changes in the liver and stomach of mice but no sign of diarrhea was observed [66].

DSP toxins are, by far, the most monitored group of lipophilic toxins using SPATT devices. SPATT technology was first applied in 2004 to the detection of DSP toxins during *Dinophysis acuminata* blooms in several locations of New Zealand [21]. Three adsorbent resins (Diaion^®^ HP20, Diaion ^®^ HP2MG and Sepabeads^®^ SP207) were evaluated for the adsorption of OA, DTX1 and PTX2 from seawater. The recovery of OA, DTX1 and PTX2 from SP207 and HP2MG was on average only 36% and 62%, respectively, of that of HP20, which was thus proved to be the most effective. Furthermore, during field trials, despite the presence of limited number of *Dinophysis* cells in the water column (~200 cells/L), significant quantities of OA (~15 ng/SPATT bag), DTX1 (~10 ng/SPATT bag), PTX2 (~75 ng/SPATT bag) and PTX2 seco acid (PTX2sa) (~10 ng/SPATT bag) could be detected in SPATT bags made with HP20 after only 3.5 h of exposure, and increased linearly with respect to exposure times (i.e., 22.5 and 42.25 h), indicating that SPATT technology had a very high sensitivity and provided the opportunity for advanced early warning periods. Even more, during a minor bloom of *D. acuminata*, SPATT bags were able to accumulate OA + DTX1 and PTX2 at levels well above the detection limits (i.e., 70 and 600 times over, respectively) one week prior to the shellfish reaching their maximum level of toxicity. It was thus concluded that SPATT technology also has the potential to provide a means of predicting the net accumulation of polyether toxins by shellfish and forecasting shellfish contamination events.

Since this first study, several in vitro and field studies have been conducted to evaluate the capacity of different adsorbent resins to accumulate DSP toxins. For instance, in 2008, Fux et al. [33] conducted both in vitro and field experiments to describe the uptake and desorption behavior of OA and DTX1 by five styrene-divinylbenzene based polymeric resins: Sepabeads^®^ SP850, Sepabeads^®^ SP825L, Amberlite^®^ XAD4, Dowex^®^ Optipore^®^ L-493 and Diaion^®^ HP20. All resins were able to accumulate OA and DTX1 (199.5 ± 31.8, 311.3 ± 60.9, 355.9 ± 49, 431.9 ± 74.1, 460.3 ± 48.1 ng/g resin for L-493, HP20, SP825, SP850 and XAD4, respectively) following a 12-h exposure to a *P. lima* culture. After 72 h of exposure, the HP20 resin accumulated the largest amount of OA and DTX1 (1607.2 ± 238.3 and 1291.3 ± 185.4 ng/g resin, respectively) compared to the other resins assessed, reaching 24 and 12% of the free OA and DTX1 contained in the cells and in the culture media. Furthermore, after an immersion in Galway Bay (Ireland), it was shown that HP20 had the ability to accumulate a significantly larger amount of OA than SP825 and SP850 (~380 ng/g resin vs. ~200 and 180 ng/g resin after a one-week deployment at 1 m depth for example), while L-493 and XAD4 showed extremely poor performances (~80 and 20 ng/g resin). In addition to OA, quantifiable amounts of DTX2 were also accumulated using the HP20, SP825L and SP850 resins (46.4 ± 8.3, 33.3 ± 6.1, 30.5 ± 11.5 ng/g resin, respectively). McCarthy et al. (2014) [43] further monitored a marine reserve in Ireland over a four-month period using two adsorbent resins, HP20 and Amberlite^®^ XAD761. OA, DTX2, PTX2 and PTX2sa were detected from SPATT extracts throughout the study period. Again, HP-20 was found to be more effective in accumulating DSP toxins than XAD761 (e.g., OA 2.5:1, HP20:XAD761). The only exception was PTX2sa for which a higher quantity per gram of resin was accumulated in XAD761 (2:1, XAD761:HP20). More recently, Zendong et al. (2014) [34] exposed Oasis^®^ HLB, HP20 and Strata-X^®^ sorbents for 24 h to seawater spiked with algal extracts containing known amounts of OA and DTX1. Although the adsorption rate of toxins on HP20 was slower than on Oasis HLB and Strata-X resins (e.g., 40% of OA adsorbed after 7 h for HP20 vs. 60 and 70% for Oasis HLB and Strata-X, respectively), HP20 and Strata-X gave somewhat higher recoveries than Oasis HLB after 24 h exposure (e.g., ~80 and 70 ng of OA per g of resin for Strata-X and HP20 vs. ~50 ng/g for Oasis HLB). Trials in re-circulated closed tanks with mussels exposed to *P. lima* and with different sorbent materials competing for toxins in the same container showed that Strata-X accumulated OA and DTX-1 faster than Oasis HLB and HP20, and to higher levels (e.g., ~7500 ng of OA per g of resin for Strata-X vs. ~6000 and ~100 ng/g for Oasis HLB and HP20, respectively). However, following a three-week field trial in Ingril lagoon (Mediterranean Sea), HP20 showed higher recoveries than the other resins (e.g., ~400 ng of OA per g of resin for HP20 vs. ~100 and 250 for Strata-X and Oasis HLB after seven days of deployment). According to the authors, this difference could be the result of the competition between resins during tank trials, which was detrimental to HP20 because of its low accumulation speed. They thus concluded that Strata-X and Oasis HLB, which are fast accumulators, would be more appropriate for daily or on-board evaluation of toxin presence, and the use of HP20 more advisable in trials with long exposure periods.

Several studies have also been conducted in order to investigate the potential use of these passive SPATT filters as an early warning system of DSP toxic dinoflagellates occurrence and thus their ability to provide a means of forecasting DSP contamination events, as suggested by the initial study conducted by MacKenzie et al. [21]. Li et al. [49] monitored DSP toxins over a 10.5-month period in shellfish farming areas in Lingshan Bay of the Yellow Sea in China. OA and DTX1 were detected in SPATT extracts (165 and 56 ng/g HP20 resin, respectively) 14 days before levels peaked in scallops. The authors thus concluded that administrators had about two weeks to warn of the potential for toxin contamination in shellfish. Even if this study is in favor of the potential use of SPATT devices as an early warning system of shellfish contamination, other studies obtained results that were in contradiction with this statement. For example, Fux et al. (2009) [35] monitored DSP toxins during four months in three shellfish production sites of the west coast of Ireland (Atlantic Ocean) using SPATT discs (HP20 resin) and toxin-free (i.e., transplanted) mussels that were replaced weekly. The toxin profiles and concentrations obtained in the SPATT and in the transplanted mussels were compared with those observed in indigenous (native) mussels from each production site as well as with the phytoplankton that was detected in the water. The accumulation rate of toxins in the indigenous mussels and in the SPATT discs showed good correlation. OA, DTX2 and PTX2 were detected in all SPATT discs (with a maximum of 5645, 617 and 1265 ng/g HP20 resin for OA, DTX2 and PTX2, respectively), even in the absence of toxin-producing phytoplankton (SPATT discs thus indicated levels of 1200–1400 and 12–25 ng/g HP20 resin for OA and PTX2, respectively). However, the authors also observed during this study that no DTX2 was detected in the SPATTs prior to the occurrence of *D. acuta* in seawater, and that DTX2 appeared in the SPATT samplers at the same time as in the mussels. Therefore, they conclude that SPATT were here not able to forecast shellfish contamination. In the same way, Rundberget et al. (2009) [36] concluded that it is difficult to recommend the passive samplers as an early warning tool, since they obtained inconclusive results during their study conducted in Norway. These authors compared the profiles of accumulated toxins in SPATT devices and toxin profiles in blue mussels (*Mytilus edulis*) with the relative abundance of toxin-producing algal species. Toxins detected on passive sampling discs correlate well with the toxin profiles in shellfish. However, during the monitoring period, even if two increases in OA/DTXs in the SPATT discs (reaching a maximum level of ~700 ng/g HP20 resin) and in *Dinophysis* cell numbers were observed some time before the levels of toxins in the shellfish increased, an increase of *Dinophysis* cell densities and OA/DTXs levels in the discs with no corresponding increase in the shellfish was also observed, suggesting that SPATT devices could lead to a false alarm. A third study weekly deployed SPATT devices (HP20 resin) in the northwestern coast of Spain for seven months, in parallel with a weekly monitoring of phytoplankton and toxin content in picked cells of *Dinophysis* and plankton concentrates [40]. Weekly adsorption of toxins in the SPATT discs included those detected in picked cells of *Dinophysis* and in the plankton concentrates, i.e., OA, DTX2, PTX2 and PTX2sa (reaching a maximum of ~4500, 1900, 2800 and 900 ng/g HP20 resin, respectively, for a deployment at 0–5 m depth for example). However, results showed that detection of *Dinophysis* populations provided earlier warning of oncoming DSP outbreaks than the SPATT, which at times overestimated the expected toxin levels in shellfish. Authors explained this observation by the fact that toxins accumulated by SPATT devices did not include biotransformation and depuration loss terms, and by the fact that accumulation of toxins not available to mussels continued for weeks after *Dinophysis* cells were undetectable and mussels were toxin-free. They thus conclude that SPATT technology may be a valuable environmental monitoring and research tool for toxin dynamics, in particular in areas with no aquaculture, but does not provide a practical gain for early warning of DSP outbreaks.

Through all the studies conducted, SPATT technology using HP20 resin was effective to detect numerous DSP toxins all over the world, with good correlation between toxin profiles obtained in the SPATT devices and presence of associated phytoplankton cells and/or their toxin profiles. OA, DTX1, DTX2, PTX2, PTX2sa and 7-epi-PTX2sa (maximum ~5600, 3500, 25,000, 900 and 9 ng/g HP20 resin, respectively), as well as an isomer of PTX11 and an isomer of PTX3, were detected in Ireland, where the use of passive sampling has also provided the capability of obtaining toxin profiles at very low depths (110 m) [33,35,37,43]. OA and DTX2 (maximum 650 ng/g HP20 resin for OA/DTXs), together with PTX2, PTX2sa and PTX12, were detected in Norway [36]. OA, DTX2, PTX2 and PTX2sa (reaching a maximum of ~4500, 1900, 2800 and 900 ng/g HP20 resin, respectively, for a deployment at 0–5 m depth for example), as well as OA-D8 and a PTX11 isomer were detected in Spain [40,50,51]. OA, DTX-1, PTX-2, PTX-2sa and 7-epi-PTX-2sa were detected in the Yellow Sea in China (maximum 165, 56, 107 and 50 ng/g HP20 resin for OA, DTX-1, PTX-2 and PTX2-sa + 7-epi-PTX-2sa, respectively) [49,52]. Finally, OA, DTX1, PTX2 and PTX2sa were detected in Nigerian coastal waters of Africa (Atlantic Ocean) (60 ng OA/g HP20 resin) [42] and in French coastal areas of the Mediterranean Sea (650, 120, 500, 100 ng/g HP20 resin, respectively) and Atlantic Ocean (130, 3, 1800, 160 ng/g HP20 resin, respectively) [23], where results also showed that toxin concentrations and profiles in SPATTs were dependent on the amount of resin used (0.3, 3, and 10 g of HP20 resin) and that SPATTs bags containing 3 g of resin appeared to be the best compromise.

A system to mimic more accurately the adsorption of toxins by shellfish in two warm-temperate estuaries of New Zealand (Rangaunu and Parengarenga harbors) was also developed [53]. SPATT bags (HP20 resin) were placed within “Clam” samplers that were designed to simulate the position of oysters on the intertidal racks and keep the SPATT bags immersed in seawater, and protected from the effects of exposure to wind and sunlight, during low tide periods. The samplers consisted of a plastic box with a hinged lid. Attached beneath the lid was a removable cage that contained the SPATT bag and on top a block of high-density polystyrene. As the tide rose, the buoyant lid opened, exposing the SPATT bag to the seawater, and when the tide ebbed, the lid closed and the SPATT bag was immersed in the remaining water in the box. Over the two summers that SPATT bags were deployed, LC–MS multi-toxin screens showed that all contained high levels of OA (33.2–591.3 ng/g HP20 resin) with trace amounts of PTX2 and DTX1 (0.2–5.6 and 0.2–2.0 ng/g HP20 resin, respectively), together with other polyether toxins (pinnatoxins and spirolides). The bulk of the OA adsorbed to the HP20 resin in the SPATT bags clearly originates from the high numbers of okadaic acid producing *P. lima* that are epiphytic upon the sea-grass leaves.

On a more technical level, a study has investigated the dynamic adsorption of DSP toxins by two polystyrenix resins, HP20 and SP700 [54]. Results of in vitro experiments showed that the difference in adsorption capacity for OA and DTX1 toxins was not determined by specific surface area, but by the pore-size distribution. Additionally, it was found that differences in affinity between OA and DTX1 for aromatic resins were a result of polarity discrepancies due to DTX1 having an additional methyl moiety. Another study has investigated the effect of seawater salinity on pore-size distribution on HP20 resin and its adsorption of DSP toxins [55]. Taking into consideration the pore size distribution and surface images, results of in vitro experiments showed that intra-particle diffusion governs toxin adsorption in seawater at high salinity (~27‰) while film diffusion mainly controls the adsorption process in seawater at medium salinity (~13.5‰). Furthermore, this study showed that molecules of OA and DTX1 are able to enter into micropores (<2 nm) and small mesopores (2–10 nm) of HP20 resin in estuarine seawater with high salinity. However, further studies are needed to clarify whether the effect of salinity on adsorption kinetics is sufficiently significant at the naturally low concentrations of toxins in seawater to play a role during daily or weekly monitoring.

Finally, some studies have also extended the concept of SPATT technology to the use of adsorbent resins (mostly HP20 resin) for active sampling of DSP toxins. This idea, introduced in 2007 by Rundberget et al. [56], consists of large-scale pumping of seawater through adsorbent resin directly in the environment during in order to harvest toxins from field populations of algal blooms. An 18-h trial during a bloom of *D. acuta* in Spain led to the isolation (after extraction of HP20 resin and purification of toxins) of OA (2.7 mg), DTX2 (1.3 mg) and PTX-2 (1.8 mg). More recently, an active sampling performed continuously over seven days in a marine reserve of Ireland led to the accumulation of high quantities of OA (~13 mg), DTX2 (~29 mg), PTX2 (~20 mg) and PTX2sa (~6 mg) using HP20 resin [43]. This method is thus effective for accumulating DSP toxins from the environment, as well as for harvesting toxins from large-scale microalgal cultures, and should also be useful for bioprospecting and isolation of bioactive natural products from marine and freshwater environments.

#### 2.1.2. Yessotoxins (YTXs)

**Figure 3:**
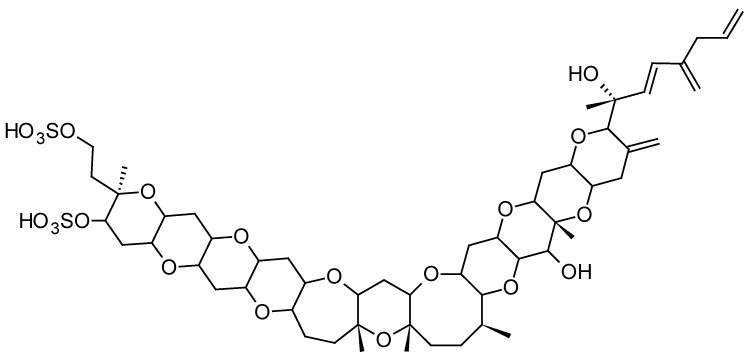
Structure of yessotoxin (YTX).

Yessotoxin (YTX) and its analogues are produced by the phytoplanktonic dinoflagellates *Protoceratium reticulatum*, *Lingulodinium polyedrum*, *Gonyaulax spinifera* and *Gonyaulax taylorii* [67,68,69,70]. Initially, YTXs were classified in the group of DSP toxins; however, since they do not share the same mechanism of action, i.e., inhibition of protein phosphatases, they have been classified as a separate group of algal toxins. YTXs are sulfur bearing polyether toxins (Figure 3) that are structurally related to brevetoxins and ciguatoxins. No reports of human poisoning induced by yessotoxins have been recorded, although YTXs contaminated shellfish are worldwide reported, sometimes at high concentrations [71]. When injected i.p. in mice, the toxicity of YTX is relatively high while oral administration of high levels of YTX did only result in some swelling of the heart muscle cells of mice [72]. A harmful algal bloom producing yessotoxin was the major causative agent of an abalone mass mortality event in California in 2011 [73].

Monitoring of YTXs using SPATT methodology is often performed at the same time as the monitoring of DSP toxins [21,35,49,57]. As for DSP toxins, Diaion^®^ HP20 resin appeared to be the most effective adsorbent for accumulation of YTX, as compared with Diaion^®^ HP2MG and Sepabeads^®^ SP207 [21]. Results of the first field trials conducted in 2004 during *P. reticulatum* blooms in New Zealand showed that the concentrations of YTX in seawater followed the relative abundance of the dinoflagellates from which they had originated [21]. The peak of the bloom (~130 × 10^3^ cells/L) was recorded thanks to the high levels of YTX detected in the SPATT bags (2–4 µg/SPATT bag/day) but was missed by the routine plankton monitoring. Similarly, in Ireland, despite the apparent absence of known YTX producing organisms in the surveyed area, YTX was detected above quantification limit in all the SPATT bags (23–347 ng/g HP20 resin) deployed during a two-month survey of McSwynes Bay (Ireland), as well as in toxin-free mussels transplanted at the beginning of the survey [35]. In the East China Sea, no YTX but homo-yessotoxin (homoYTX), was detected in both SPATT discs (1.78–10 ng/g HP20 resin) and mussels during a 10-day survey [57]. Finally, in a shellfish farming area in Lingshan Bay of the Yellow Sea (China), traces of YTX (no quantifiable levels) was detected among other toxins (OA, DTX1, PTX2) using SPATT technology (HP20 resin), however, no YTX was clearly detected in neighboring scallops [49].

#### 2.1.3. Azaspiracid Shellfish Poisoning (AZP) Toxins

**Figure 4:**
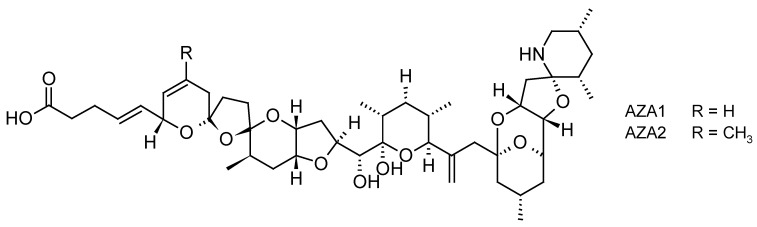
Structures of azaspiracids 1 and 2 (AZA1, AZA2).

Azaspiracid poisoning (AZP) is a fairly newly identified human toxic syndrome associated with shellfish that was described 20 years ago [74]. The group of AZP toxins, known as azaspiracids (AZAs) (Figure 4), differ from any of the previously known nitrogen-containing toxins found in shellfish or dinoflagellates since they have unique spiro ring assemblies [75]. AZAs are produced by some species of the planktonic dinoflagellate of the Amphidomataceae family, i.e., *Azadinium spinosum, Azadinium poporum* and *Azadinium dexteroporum and Amphidoma languida* [76]. The mechanism of action of AZAs is not yet fully understood and remains elusive despite numerous in vitro and in vivo toxicological experiments aiming at determining its biological target [77,78]. However, it appears that for example, AZAs affect the actin cytoskeleton and therefore some cytoskeleton-dependent functions, such as maintenance of cell shape or adhesion [79]. Following the consumption of contaminated shellfish, gastrointestinal symptoms appear within hours of ingestion such as nausea, vomiting, severe diarrhea, and stomach cramps, similar to those observed during DSP [80]. However, no fatal cases have been reported in humans. Negative effects on marine fauna were also reported with AZA1 inhibiting the development of Medaka embryos [81] as well as impacting the feeding behavior of blue mussels by decreasing filtration and increasing pseudofeces production [82].

As for YTXs, monitoring of AZAs using SPATT methodology is often performed as part of the monitoring of DSP toxins [33,34,35,36]. During their field trials in the northwestern coast of Ireland, Fux et al. (2008) [33] observed the accumulation of AZA1 and AZA2 at quantifiable levels in SPATT devices made with HP20 resin (740.5 and 219.9 ng/SPATT disc), as well as the accumulation of AZA3 at traces levels (52.3 ng/SPATT disc). In a second study, Fux et al. (2009) [35] deployed SPATT filters (HP20 resin) during a severe toxic event of AZAs and were able to confirm the efficacy of these passive samplers to accumulate AZA1, AZA2 and AZA3 (reaching a maximum concentration of 6816, 3151 and 468 ng/g HP20 resin). However, authors concluded that, in this case, SPATT did not enable the forecasting of shellfish contamination, since the accumulation of AZAs in the SPATTs followed the accumulation pattern that was observed in mussels. In Norway, Rundberget et al. (2009) [36] showed that blue mussels contained AZA1, AZA2, AZA3 and AZA6. SPATT bags (HP20 resin) deployed near these shellfish were able to accumulate AZA1 and AZA2 (in a ratio of 5:1) but no AZA3 and AZA6 that are suspected to be not produced by dinoflagellates but to be the result of transformation of AZA1 and AZA2 in shellfish. More recently, a new azaspiracid, named AZA-59, was isolated from four strains of *A. poporum* from Puget Sound (Washington State, USA). The presence in the field of AZA-59 was confirmed at trace levels using SPATT devices (HP20 resin) deployed at several stations along the coastlines of Puget Sound [59].

As for other toxins, even though HP20 resin is the most widely used resin, other sorbents also proved effective for the accumulation of AZAs in SPATT format. For example, Sepabeads^®^ SP700 was able to retain low concentrations of AZA1, together with other lipophilic toxins, during a monitoring of *Alexandrium ostenfeldii* and associated spirolides toxins in Ireland [58]. Furthermore, Oasis^®^ HLB, and Strata-X^®^ sorbents have shown the capacity to accumulate AZA1 and AZA2, with adsorption rates higher than the one of HP20 resin (e.g., 40% and 50% for HLB and Strata-X vs. 30% for HP20 resin after 7 h of exposure to spiked seawater), however, after 24 h of exposure, less levels of AZA-1 were recovered from these resins (~40 and 50% for HLB and Strata-X vs. 70% for HP20) [34].

#### 2.1.4. Cyclic Imines (CI)

**Figure 5:**
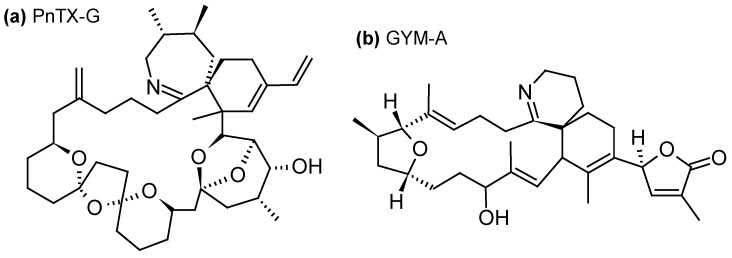

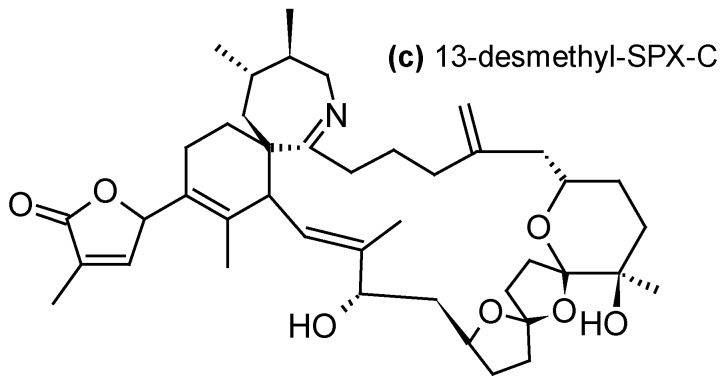
Structures of (**a**) pinnatoxin-G (PnTX-G), (**b**) gymnodimine A (GYM-A), and (**c**) 13-desmethyl spirolide-C (13-desmethyl-SPX-C).

Since their description in mid-1990s, cyclic imines (CIs) form a group of marine toxins with no proven records of acute toxicity in humans. Nevertheless, their increasing occurrence in warming oceanic environments and potent toxicity warrant vigilance while monitoring CI levels in marine products [83]. CIs group can be divided into three subgroups: the pinnatoxins (PnTXs) (Figure 5a), produced by the dinoflagellate *Vulcanodinium rugosum* [84]; the gymnodimines (GYMs) (Figure 5b), produced by the dinoflagellates *Gymnodinium* cf. *mikimotoi* [85], *Karenia selliformis* [86], *Alexandrium peruvianum* [87] and *A. ostenfeldii* [88]; and the spirolides (SPXs) (Figure 5c), produced by the dinoflagellates *A. ostenfeldii* [89] and *A. peruvianum* [90]. CIs are macrocyclic compounds with imine (carbon-nitrogen double bond) and spiro-linked ether moieties. The toxicity of CIs is mainly due to their effect on the cholinergic system, through their interaction with nicotinic acetylcholine receptors located at the neuromuscular junction [83]. The mechanism of action is not yet completely understood, but i.p. injection of GYMs or SPXs caused very rapidly acute toxicity and death in mice [91,92]. For this reason these toxins have been classified as fast-acting toxins. Toxic effects of *V. rugosum* on larvae of sea urchins, oysters and *Artemia* were also reported [93].

Using SPATT devices filled with HP20 resin, 13-desmethyl-SPX-C was detected in northwestern and southwestern coasts of Ireland (0.32–24 ng/g HP20 resin) [35,44], in warm-temperate estuaries of the Northland of New Zealand (0.1–0.2 ng/g HP20 resin) [53], in Norway (0.3–4.8 ng/g HP20 resin) [39] and in French coastal areas of the Atlantic Ocean (trace amounts) [23]; 20-methyl-SPX-G was detected in Norway (9.8–55 ng/g HP20 resin) [36,39] and in southwestern coast of Ireland (2.5–2.24 ng/g HP20 resin) [44]; and 13,19-didesmethyl-SPX-C and iso-SPX-C were detected in Norway (0.5–77 and 0.1–0.6 ng/g HP20 resin) [39]. Furthermore, the use of Sepabeads^®^ SP700 resin allowed the detection of 13-desmethyl-SPX-C and 20-methyl-SPX-G in south coast of Ireland, even when no *A. ostenfeldii* cells were detected in the environment. The maximum combined SPXs concentration (2.5 ng/g resin/day) coincided with the maximum *A. ostenfeldii* cell concentration observed in the study site [58]. 13-desmethyl-SPX-C was also successfully adsorbed on Oasis^®^ HLB and Strata-X^®^, with similar recoveries than with HP20, but also Amberlite^®^ XAD761 resins with lower efficiency than with HP20 (ratio 4.7:1 HP20:XAD761) [34,44].

In the same way, using HP20 resin, SPATT devices allowed the detection of PnTX-D, PnTX-E and PnTX-F in warm-temperate estuaries of the Northland of New Zealand (1.2–3.0, 45.6–226.0 and 2.1–106.6 ng/g HP20 resin, respectively) [53]; as well as PnTX-G in Norway (0.5–15 ng/g HP20 resin) [39], in Catalonia (northwestern Mediterranean Sea) (0.28–0.93 ng/g HP20 resin) [31], in southwestern coasts of Ireland (0.1–0.45 ng/g HP20 resin) [44] and in French coastal areas of the Mediterranean Sea (trace levels) [23]. As for 13-desmethyl-SPX-C, PnTX-G was also successfully adsorbed using Oasis^®^ HLB, Strata-X^®^ and Amberlite^®^ XAD761 resins with lower efficiencies than with HP20 (~55% of toxin recovery for HLB and Strata-X vs. 95% for HP20, and a ratio 1.45:1 HP20:XAD761) [34,44].

Finally, only one field study has documented the detection of GYM-A (no quantification) using SPATT bags filled with HP20 resin and deployed in the Zhejiang Coast along Eastern China, in parallel with its detection in neighboring mussels [57].

#### 2.1.5. Ciguatoxins (CTXs)

**Figure 6:**
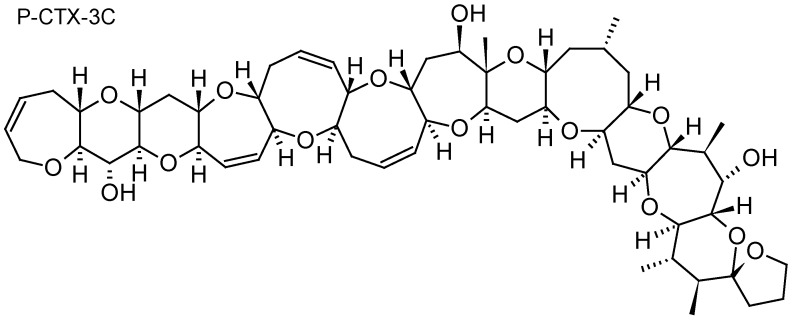
Structure of pacific-ciguatoxin-3C (P-CTX3C).

Ciguatera fish poisoning (CFP) is a food-borne illness endemic in tropical and subtropical coral reef regions of the world (Pacific Ocean, Indian Ocean and Caribbean Sea). Ciguatera is caused by the consumption of tropical coral reef fish contaminated with ciguatoxins (CTXs) produced by dinoflagellates belonging to the genera *Gambierdiscus* and *Fukuyoa* [94]. CTXs are lipid-soluble polyether compounds consisting of 13 to 14 rings fused by ether linkages into a most rigid ladder-like structure (Figure 6). To date, more than 40 congeners have been isolated from *Gambierdiscus* cultures or contaminated fish, with structural differences between CTXs from the Pacific (P-CTX), Caribbean (C-CTX), and Indian Ocean (I-CTX) [95]. CTXs stimulate Na^+^ entry through specific binding to site 5 of the voltage-gated Na^+^ channel, thus inducing many effects at the cellular and physiological levels, such as membrane excitability, release of neurotransmitter, axonal and Schwann edema, increase of intracellular calcium, and blockage of voltage potassium channels [95]. General (e.g., weakness, joint pains, myalgia, headache, dizziness, itching), digestive (e.g., nausea, vomiting, diarrhea, abdominal pain, cramps, dehydration), cardiovascular (e.g., low arterial pressure, irregular heartbeat, bradycardia), and neurological (e.g., dysesthesia, temperature reversal, paresthesia, superficial hyperesthesia) symptoms are commonly reported during CFP outbreaks [96]. CTXs have been shown to induce behavioral abnormalities in fish following feeding experiments with toxic cells or flesh [97,98], cause fish mortality through water exposure [99], and result in developmental toxicity in finfish [100,101].

The first study to investigate the efficacy of SPATT devices using HP20 resin for the detection of dissolved CTXs in *Gambierdiscus* cultures was the one by Caillaud et al. (2011) [30]. These authors were able to confirm the ability of HP20 resin to recover both dissolved P-CTX1B standard (~90%) and dissolved fraction of CTX-like compounds produced by a *G. pacificus* strain. More recently, in a field study conducted in a CFP hotspot of French Polynesia, Roué et al. (2017) [32] were able to validate for the first time the relevance of SPATT technology for the routine monitoring of *Gambierdiscus* and *Fukuyoa* toxins in the field. Indeed, these authors were able to demonstrate the effectiveness of SPATT devices (HP20 resin) for the detection of P-CTX3B, P-CTX3C and iso-P-CTX3B/C in Anaho Bay (Nuku Hiva Island, Marquesas archipelago) (53.64 ± 13.69 ng P-CTX3C equiv/g HP20 resin), a site known for its high prevalence of toxic marine products (both fish and invertebrates) and where significant amounts of *Gambierdiscus* cells are consistently detected [102,103]. These results suggest that SPATT technology could be used to detect the presence of toxic *Gambierdiscus* cells through the detection of CTXs.

### 2.2. Hydrophilic Toxins

Hydrophilic toxins are produced by freshwater and marine microorgansims (cyanobacteria and microalgae). As lipophilic toxins, they can be very harmful to humans, but also wildlife, and regulatory limits have been set for some of them. For example, more than a dozen countries have developed regulations or guidelines for microcystins in drinking water and recreational waters. Given the usefulness of SPATT technology to detect a wide range of lipophilic toxins, the extension of this passive monitoring system to the detection of more polar water-soluble toxins has also been assessed in the frame of several studies. SPATT devices, made with various adsorbent substrates, have thus proven to be effective for the detection of domoic acid (DA), saxitoxins (STXs) and derivatives (GNTXs, C-toxins), anatoxins (ANTXs) and mycrocystins (MCs) (Table 2). The major in vitro and field studies conducted are reviewed and detailed below.

#### 2.2.1. Amnesic Shellfish Poisoning (ASP) Toxins

**Figure 7:**
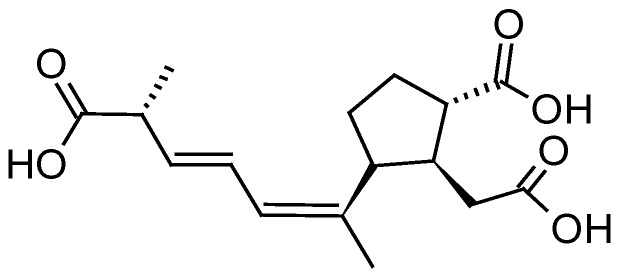
Structure of domoic acid (DA).

Domoic acid (DA) (Figure 7) is a crystalline water-soluble acidic amino acid naturally produced by several diatom species of the genus *Pseudo-nitzschia* [111]. This potent neurotoxin is responsible for amnesic shellfish poisoning (ASP) cases in humans that consist of gastrointestinal disorders, confusion, disorientation, seizures, permanent short-term memory loss, and even death in severe poisoning cases [112]. DA acts as a glutamate agonist, causing excitotoxicity in the vertebrate central nervous system and other glutamate receptor-rich organs [112,113]. DA also provoked alteration of phagocytic activity of hemocytes along with their decreased in *Crassostrea gigas* [114].

The applicability of SPATT technology to detect the DA was tested using four resin types: Diaion^®^ HP20, Sepabeads^®^ SP700, SP207 and SP207SS [45]. All resins successfully adsorbed DA, HP20 being the less efficient (HP20 < SP700 < SP207 and SP207SS). However, compared with HP20, the three other resins were relatively aggressive in their retention of DA, with DA recovery efficiencies from the SPATT bags comparatively very low (2–11%). SPATT devices filled with HP20, SP700 and SP207 resins were further used in a 17-month monitoring study during which SPATT units were weekly deployed in Monterey Bay, California (USA, Pacific Ocean) [45]. The three resins were able to signal the presence of DA over periods in which resident toxigenic *Pseudo-nitzschia* species were not detected by weekly phytoplankton observation, with HP20 being the most effective (maximum 25 ng DA/g HP20 resin). Furthermore, during this period, two toxic *Pseudo-nitzschia* bloom events occurred. SPATT filled with HP20 resin signaled the presence of DA three and seven weeks before the recognition of bloom conditions by traditional monitoring techniques, and seven and eight weeks before shellfish became toxic. In another study by Herman et al. (2010) [104], SPATT bags containing SP700 resin were deployed in Loch Ewe in Scotland on a weekly basis in conjunction with sampling of phytoplankton. Results confirmed that SP700 resin has the ability to trace the variations of concentration of DA dissolved in the water column (maximum 20 ng/g resin), even when levels of toxic phytoplankton being present in the water column are low. Finally, more recently, during a long-term survey of the land–sea interface of San Francisco Bay (California) based on particulate grab samples, SPATT samplers (HP20 resin) successfully detected DA (maximum 1950 µg/kg HP20) even if *Pseudo-nitzschia australis* was not apparent [105].

#### 2.2.2. Paralytic Shellfish Poisoning (PSP) Toxins

**Figure 8:**
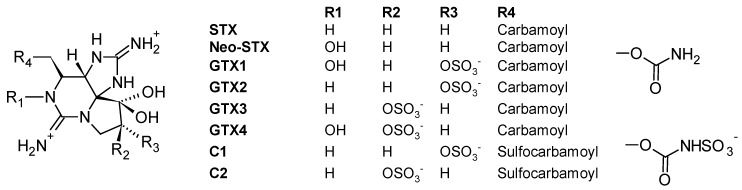
Structures of saxitoxin (STX), neo-saxitoxin (neo-STX), gonyautoxins 1 to 4 (GTX1, GTX2, GTX3, GTX4) and C-toxins 1 and 2 (C1, C2).

The PSP toxins form a group of closely related tetrahydropurine compounds including saxitoxin (STX) and neoSTX, as well as gonyautoxins (GTXs) and C-toxins (C1-4) (Figure 8) [115]. They are produced by both marine dinoflagellates (*Alexandrium*, *Gymnodinium* and *Pyrodinium*) and freshwater or brackish cyanobacteria (*Anabaena*, *Cylindrospermopsis*, *Aphanizomenon*, *Planktothrix* and *Lyngbya*) [115]. The toxicity of PSP toxins is caused by a high affinity inhibition of voltage-gated sodium channels present on the extracellular membrane of excitable cells like muscle and nerve cells, resulting in a reduced action potential. At low levels of exposure, these toxins can cause numbness of the fingers and extremities, tingling, nausea and vomiting; but at higher doses, they can result in muscular paralysis and death by respiratory paralysis and cardiovascular shock [116]. STXs have been associated with numerous human intoxications resulting in numbness, complete paralysis and even fatalities. Regarding ichtyotoxic effects, gill lesions preceding mortality were observed in Chilean farmed salmon during *Alexandrium catanella* blooms and *Alexandrium monilatum* [117].

The efficacy of SPATT technology for the monitoring of PSP toxins was initially tested during a 17-month field survey with approximately weekly SPATT deployments in Monterey Bay, California (USA, Pacific Ocean) [45]. During the study period, blooms with low-level of *Alexandrium catenella* cells occurred, yet, SPATT devices (HP20 resin) afforded the direct detection of PSP toxins including STX in the water column (maximum 0.04 ng PST/g HP20 resin). In another in vitro study by Rodriguez et al. (2011) [106], dialysis bags holding SP700 resin or a computationally designed polymer (CDP) based on the functional monomer ethylene glycol methacrylate phosphate (EGMP) were deployed in PSP toxins-spiked seawater and in cultures of *A. tamarense*. Both resin substrates successfully adsorbed STX, neo-STX, GTX1, GTX2, GTX3, GTX-4, C1 and C2 for a limited period of time (3–7 days) (60, 81, 173, 176 and 427 ng/g CDP for GTX2-3, C1-2, STX and GTX1-2, respectively, and 6, 16, 68, 183 and 194 ng/g SP700 resin for GTX2-3, STX, neoSTX, C1-2 and GTX1-4, respectively, after seven days of deployment in *A. tamarense* cultures), demonstrating that either CDP or SP700 resin could be used in SPATT deployments for the monitoring of PSP toxins in the water column. However, authors noted that a major limitation of both CDP and SP700 resin was the fast toxin desorption from the resins when these latter were deployed in toxin-free seawater (loss of 59%, 68%, 69%, 79% from CDP for neoSTX, GTX1-4, STX, GTX2-3 and C1-2, respectively, and loss of 78%, 80%, 82%, 83% and 91% from SP700 resin for GTX1-4, neoSTX, GTX2-3, STX and C1, respectively, after four days in seawater), a likely result of the high aqueous solubility of these compounds. It was thus concluded that in this case, SPATT deployment in the water should not exceed seven days to avoid toxin loss due to desorption.

#### 2.2.3. Microcystins (MCs) and Nodularins (NODs)

**Figure 9:**
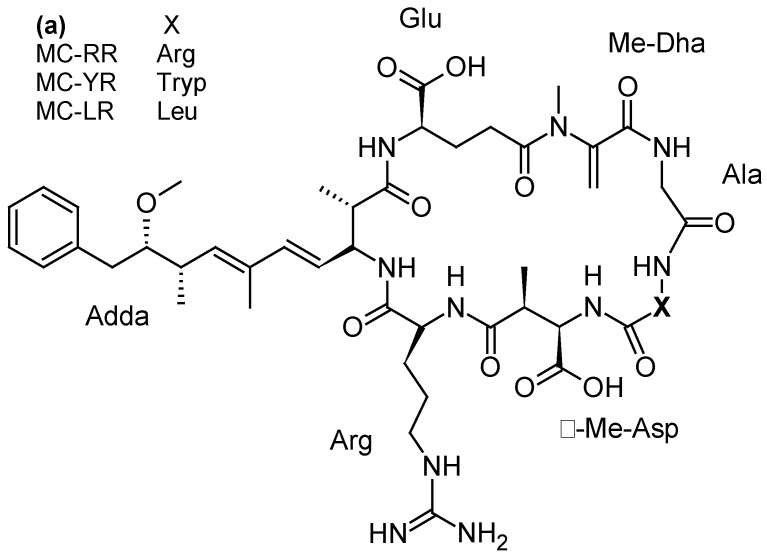

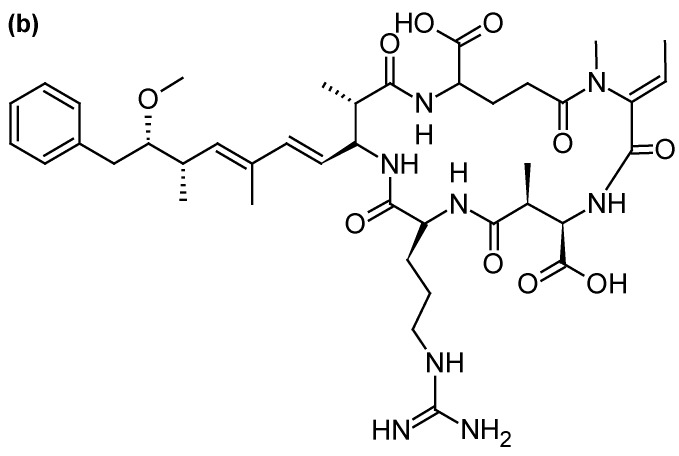
Structures of (**a**) microcystins –RR, –LR, and –YR (MC-RR, MC-LR, MC-YR) and (**b**) nodularin (NOD).

Microcystins (MCs), one of the most abundant groups of cyanotoxins worldwide, are cyclic peptides with different levels of toxicity and varying by degree of methylation, hydroxylation, epimerization, and peptide sequence. The most common microcystins are MC-LR, MC-RR and MC-YR (Figure 9a). MCs are produced by freshwater cyanobacteria, mainly *Microcystis* but also *Oscillatoria, Nostoc, Anabaena* or *Anabaenopsis* genera [3]. Their mode of action is associated with the specific inhibition of protein serine/threonine phosphatases (PP1 and PP2A), altering phosphorylation of cellular proteins involved in signal transduction [118]. At high levels of exposure (representative of acute intoxication), MC-LR produces a cascade of events (cytoskeleton alterations, lipid peroxidation, oxidative stress, apoptosis) leading to centrolobular toxicity with intrahepatic hemorrhagic areas due to damage of sinusoidal capillaries. At low doses (typical of long-term exposure), phosphatases inhibition induces cellular proliferation and hepatic hypertrophy [118]). Numerous animal and human intoxications by MCs have been reported [119]. Most of the human poisonings were limited to gastroenteritis but, on one occasion, MCs-contaminated water used for hemodialysis caused the death of 60 patients at the Brazilian dialysis center of Caruaru in 1996 [120]. A severe liver failure case was also reported from a child whose family was exposed to MCs recreational exposure during algal bloom occurring in a Carrasco beach (Uruguay) [121]. Furthermore, the consumption of crops grown with MC-contaminated irrigation water can pose a risk to health and food security [122].

Nodularin (NOD) is a cyclic peptide structurally close to MCs (Figure 9b), produced by the planktonic cyanobacterium *Nodularia spumigena* occurring in brackish waterbodies [123]. So far, 9 variants of this water soluble and stable toxin have been identified [3]. Like MCs, NODs are hepatotoxins inhibiting protein phosphatases 1 and 2A and can act as a liver tumor initiator and promoter [124]. Nodularin was involved in death of animals by severe liver hemorrhages [125]. Furthermore, it could be accumulated in shellfish and other seafood, which can become hepatotoxic and hazardous to human health [126]. However, no human intoxication with NODs has been reported so far.

The first study to demonstrate the excellent adsorption capacity of SPATT filters towards MCs was conducted by Miller et al. (2010) [38] in both fresh and salt waters of California. SPATT bags filled with HP20 resin were placed in laboratory conditions into subsamples of concentrated water/*Microcystis* mixtures from Pinto Lake (freshwater), known to be a hotspot of toxic cyanobacterial blooms. An adsorption of 100% of free MCs was observed in less than 24 h. Furthermore, a higher sensitivity of SPATT for MCs detection than intermittent grab samples was noted (100 ng/g HP20 resin detected with SPATT bags vs. no MCs detected using grab sampling). SPATT bags were then deployed in Pinto Lake but also at the marine outfalls of the Pajaro and Salinas Rivers and in the ocean in Monterey Bay where sea otters were found dead poisoned by MCs. MCs were successfully detected in SPATT deployed at the marine outfalls of the Pajaro and Salinas Rivers. A second study conducted by Kudela et al. in 2011 [46], also in Pinto Lake, compared the suitability of SPATT sampling with that of traditional grab sampling for the detection of MCs*.* HP20 exhibited excellent adsorption and recovery characteristics for MC-LR (1.01–895.4 ng/g HP20 resin/day), but also MC-YR, MC-LA, and MC-RR. SPATT proved to be a robust technique, detecting MCs during every deployment in contrast to the grab samples (for which 42% were found below the limit of detection, using liquid chromatography–mass spectrometry for microcystin-LR). Finally, a third study, conducted by Gibble et al. (2013) [41], surveyed 21 freshwater, estuarine, and marine locations in and around Monterey Bay for MCs presence at the land–sea interface using SPATT technology (HP20 resin). During the first year of the monitoring, MCs were detected in 15 out of 21 locations. The monitoring of four toxic sites was continued for two years with the weekly deployment of SPATT devices. Results highlighted the widespread occurrence of MCs at low to moderate levels throughout all major watersheds in the Monterey Bay area (mean levels from 0.59 to 7.91 ng/g HP20 resin, with a maximum measured at 104.31 ng/g). Here, SPATT technology allowed the time integrative survey of MCs simultaneously at different locations, providing more than a snapshot of information such as would have been obtained in the context of an intensive survey. More recently, SPATTs indicated MCs were prevalent throughout lentic waterbodies in Coastal Southern California (0.5–100.8 ng/g resin), and that traditional discrete samples underestimated the presence of MCs [107]. Indeed, MCs were detected at only 29% of sites based on discrete sample results, while time-integrated SPATT results detected dissolved MCs at 83% of sites. A similar result was obtained by Peacock et al. (2018) at the land–sea interface of San Francisco Bay (California) [105]. SPATT samplers (HP20 resin) indicated that there is a chronic, system-wide dissolved microcystins exposure to the food web year-round (maximum 25.5 µg/kg HP20). In contrast, particulate grab (filter) samples underrepresented the total amount of MCs in the water. Finally, the applicability of Sepabeads^®^ SP700 resin to adsorb MCs from *Microcystis aeruginosa* cultures was also examined by Zhao et al. (2013) [108], in comparison with HP20 resin. When SPATT bags were deployed in seawater spiked with MCs, the adsorption equilibration times for MCs were 30 and 15 min for HP20 and SP700 resins, respectively, showing that SP700 adsorbed MCs from water a little bit faster than HP20. However, better recoveries of MCs were observed from HP20 compared to SP700 (for example, 91.5% vs. 78.1% of MC-LR with HP20 and SP700, respectively). When SPATT bags were deployed for seven days in cyanobacterial cultures, the amounts of MCs adsorbed by HP20 was higher than that by SP700 (2.2 vs. 1.4 µg MC-LR/g resin for HP20 and SP700, respectively). Taking both adsorption and desorption behavior into consideration, authors finally recommended HP20 resin as an adsorbent for SPATT monitoring of MCs in freshwater.

One study has reported the detection of NOD in the periphyton of Lake Tikitapu in New Zealand, using SPATT bags made with HP20 resin (maximum 0.77 µg/g HP20 resin, 4.2 ng/SPATT bag) [109].

#### 2.2.4. Anatoxin (ANTX)

**Figure 10:**
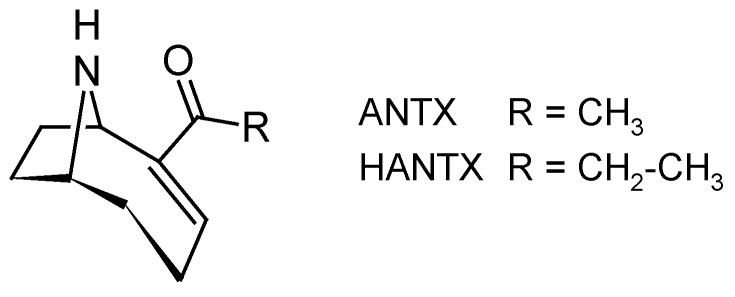
Structures of anatoxin-a (ANTX) and homoanatoxin-a (HANTX).

Cyanobacteria are known to produce diverse neurotoxins, such as PSP toxins (see Section 2.2.2) or anatoxin-a (ANTX) and homoanatoxin-a (HANTX) (Figure 10). ANTX and HANTX are unusual alkaloid, bicyclic secondary amines, usually produced by freshwater cyanobacteria belonging to several genera such as *Anabaena*, *Aphanizomenon* and *Planktothrix* [3]. Recently, HANTX was also identified from mats of a marine cyanobacterium in the genus *Hydrocoleum* [127]. ANTX and HANTX are potent agonists of the muscular and neuronal nicotinic acetylcholine receptor. Their irreversible binding to this receptor causes the sodium channels to open and a constant influx of sodium ions into the cells [128]. Overstimulation of the muscle cells occurs as a result of membrane depolarization and desensitization. When respiratory muscles are affected, the lack of oxygen in the brain may lead to convulsions and finally to death by acute asphyxia. ANTX has been responsible for various animal poisonings (dogs, cattle) resulting in vomiting, convulsion and respiratory arrest [129,130], but no human poisonings have been reported yet.

The potential of SPATT technology for collecting and concentrating ANTX and HANTX in river water was evaluated by Wood et al. in 2011 [47]. First, 15 adsorbent substrates were assessed for their ability to bind ANTX in a SPE (solid phase extraction) format. Nine substrate types, including powdered activated carbon (PAC G-60), polymeric resin (Strata-X), and strong cation exchange (MCX, Strata-X-C, AG 50W-X8 and X9) and weak cation exchange (WCX, Strata-X-CW, Amberlite IRP-64) resins, retained high proportions of applied ANTX (>70%). HP20 resin exhibited average retention of the applied ANTX (<50%). Four substrates (PAC G-60, Strata-X, AG 50W-X4 and Amberlite IRP-64) were then selected for a 24-h trial in a SPATT bag format in the laboratory. The greatest adsorption of applied ANTX was observed onto PAC G-60 (28%) and Strata-X (27%). A 3-days field study in a river containing toxic benthic cyanobacterial mats was then undertaken using PAC G-60 and Strata-X SPATT bags. ANTX and HANTX were detected at low levels in all SPATT bags (maximum 15 and 300 ng/g resin for ANTX and HANTX, respectively). Surface grab samples were taken throughout the field study and ANTX and HANTX were only detected in one of the water samples, highlighting the limitations of this currently used method. Both Strata-X and PAC G-60 were found to be effective absorbent substrates, confirming that SPATT technology has the potential to be integrated into current cyanobacterial monitoring programs for detection of ANTX and HANTX contamination in water. In a more recent study, the potential of SPATT devices filled with HP20 resin to monitor ANTX was confirmed during an extensive survey of the South Fork Eel River (California), with the detection of up to 900 ng ANTX/mg HP20 [110].

#### 2.2.5. Maitotoxins (MTXs)

**Figure 11:**
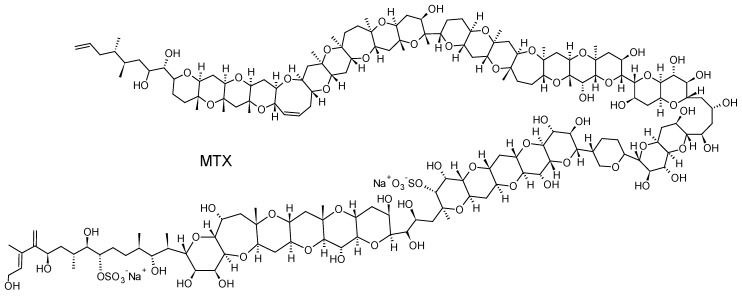
Structure of maitotoxin (MTX-1).

*Gambierdiscus* and *Fukuyoa* spp. dinoflagellates are also known to produce maitotoxins (MTXs), some compounds with extremely potent hemolytic and ichthyotoxic activities [131]. MTX is the largest non-polymeric marine toxin identified to date, consisting of a ladder-shaped cyclic polyether that is composed of 32 fused ether rings (Figure 11). Three other MTX analogs, MTX2, MTX3 and MTX4, whose structures are currently unknown, have also been isolated from *Gambierdiscus* spp. strains in culture [132,133]. MTX causes a rapid influx of external Ca^2+^ and a steep increase of intracellular Ca^2+^ concentration in a wide variety of cells. The Ca^2+^ influx elicited by MTX leads to numerous secondary events, including: depolarization in neuronal cells, phosphoinositide breakdown, smooth muscle contraction, induction of acrosome reaction in sperm, secretion of neurotransmitters, hormones and inflammatory intermediates, formation or activation of large cytolytic/oncotic pores [133]. Their implication in CFP cases is, however, currently not firmly confirmed even if a recent study has suggested that MTX could play a role if gut and liver tissues of contaminated fish are consumed [134].

The first study to investigate the efficacy of SPATT devices using HP20 resin for the detection of dissolved MTXs in *Gambierdiscus* cultures was the one by Caillaud et al. (2011) [30]. These authors were able to confirm the ability of HP20 resin to recover both dissolved MTX standard (~66%) and dissolved fraction of MTX-like compounds produced by a *G. pacificus* strain. More recently, Roué et al. (2017) [32] were able to show the potential of HP20 resin to accumulate putative MTX3. MTX3 seems to be ubiquitous within *Gambierdiscus* and *Fukuyoa* genera, suggesting its potential use as a biomarker of the occurrence of *Gambierdiscus* cells in the natural environment, regardless of their toxic potential [133,135,136]. Since this study also demonstrated the efficacy of HP20 resin to adsorb P-CTXs, it was suggested that SPATT technology could be used to detect the presence of *Gambierdiscus* cells through the detection of putative MTX3, and more specifically the presence of toxic cells through the simultaneous detection of CTXs.

## 3. Implications in Terms of Monitoring of Emerging Toxic Risks and Reinforcement of Risk Assessment Programs

The number of toxic bloom reports has increased in recent years in several aquatic areas of the planet, in both marine and freshwater environments, and will likely continue to rise in the next decades in conjunction with anthropogenic pressures and global change [137,138]. The proliferation of toxic microorganisms (mostly marine microalgae and freshwater cyanobacteria) in aquatic habitats (lakes, rivers, estuaries, and oceans) makes them important causes of water and foodborne intoxications, representing central issues in food safety, public health and human well-being [1,139]. The main health problems associated with freshwater toxins come from drinking water, recreational activities, freshwater aquaculture and shellfish that receive contaminated land waters. Marine toxins are harmful for humans through their bioaccumulation in seafood products (fish and invertebrates) and, in a lesser extent, through direct contact and inhalation of aerosols. Many countries worldwide thus maintain routine monitoring programs in order to forecast toxic bloom events in aquatic environments and to prevent associated health impacts.

Biotoxin monitoring for the protection of human health requires a reliable resource that can be sampled consistently and easily for monitoring purposes [45]. However, the collection of sentinel shellfish/fish or HABs cells presents several limitations [21]. SPATT technology has thus been proposed as a supplementary method that directly measures toxins dissolved in the water column, in order to overcome some of the issues associated with classic monitoring of aquatic toxins. Since 2004 [21], numerous studies have emphasized the potential of SPATT technology to support a more holistic regulatory approach, since its relevancy encompasses both lipophilic and hydrophilic toxin exposure, as reviewed in the present paper. These passive samplers have shown the advantage of providing a spatially and temporally integrated response, a strategy based on SPATT technology and downstream analyses such as LC-HRMS multi-toxin screenings [48] thus constitutes a relevant approach to better assess and manage the risks posed by HABs development in aquatic trophic webs.

Of the numerous adsorbent substrates tested to date, the aromatic resin DIAON^®^ HP20 is the one for which the largest amount of data are available so far, and has proven to be the most versatile adsorbent substrate both in vitro and in the field. Indeed, the HP20 resin, which has the advantage to be inexpensive [45], has proven particularly efficient in detecting a wide range of marine and freshwater biotoxins, both lipophilic (OA, DTXs, PTXs, YTXs, AZAs, SPXs, PnTXs, GYMs, CTXs) and hydrophilic (DA, STXs, GTXs, C-toxins, MTXs, MCs and, to a lesser extent, ANTX), as detailed previously in this review. The use of SPATT devices filled with HP20 resin could thus simplify simultaneous deployment in a wide range of environments, when multiple toxins are being targeted. Indeed, various HABs species could co-exist in aquatic ecosystems, emphasizing the risk of simultaneous accumulation of associated biotoxins in neighboring shellfish/fish. Moreover, recent studies have allowed the assessment of new toxin groups, e.g., AZAs [75], and of previously unreported toxins in aquatic environments, e.g., MCs in marine coastal waters [38,41] and CTXs in temperate areas [18]. The use of SPATT technology, and especially HP20 resin, could thus be very useful to monitor simultaneously numerous toxins, and to track microbial metabolites that are not usually searched, in order to prevent and manage emerging risks in aquatic environments. Especially as the simplicity, low-cost and logistical advantages (e.g., storage and transport) offered by these easy-to-use passive samplers make SPATT technology a valuable tool well adapted to the survey of areas difficult to access, such as remote islands from South Pacific Ocean for example, or areas where shellfish/fish resources are limited or not easy to collect. The SPATT technique is also well suited for the survey of widely dispersed sampling locations, providing the means to map the spatial extent of contamination risk and to track the geographical and temporal progression of blooms. Future studies in this direction would be helpful to confirm this potential application of SPATT devices. 

Further calibrations of SPATT devices would, however, likely be required before this technology could be fully integrated into existing monitoring programs [24]. Indeed, several designs have been used for the conception (construction, type of adsorbent, resin load) and field deployment of SPATT devices [25]. Furthermore, for each class of aquatic toxins, chemical extraction protocols for desorption of toxins from resins are not always standardized, leading to various percentages of recovery. To date, there is no general consensus in the literature about the optimal deployment time for SPATT devices in the field. In fact, passive samplers were generally deployed on a weekly basis [21,31,35,40,42,46,50,51,58], but sometimes, the time of deployment was reduced to few hours [32,37,47,52,53] or extended to a month [34,41,43,44,49]. So, the deployment duration varies and generally depends on the research or monitoring program design (e.g., short- or long-term monitoring, adsorbent substrate used, toxin targeted, …) together with the feasibility on the field (e.g., duration of the field mission, accessibility of the monitored area, hydrodynamism of the monitored area, …) [25]. Another disadvantages of SPATT technology is that toxin levels retained on SPATT devices are reported in relative units (expressed in ng toxin/SPATT or ng toxin/g of resin) and cannot be readily converted into quantitative toxin concentrations in the environment [46,52]. If the kinetics are known, it is possible to directly relate, or calibrate, passive sampler concentrations to ambient water concentrations [25]. However, even if some studies demonstrate that the uptake and offload kinetics for various toxin and resin combinations can be quantitatively derived, SPATT kinetics are still generally determined empirically and, as with other passive samplers, quantitative models necessary for calibrating passive samplers to environmental concentrations are generally lacking [25]. In any case, the saturation limit of the adsorbent substrate for a given toxin, i.e., the maximum concentration of a toxin likely to be retained on a given load of resin following a given time of exposure, should also be taken into consideration to avoid any potential under-estimation of the concentration of toxin circulating in seawater. However, SPATT adsorption efficiencies and saturation limits in the field cannot be determined solely by laboratory trials [25,46] as numerous environmental factors could have an influence on the adsorption of toxins by resins, such as water salinity as demonstrated by Fan et al. (2014) [55], but also variable temperature and water chemistry, variable hydrodynamics, variable ambient concentrations of toxins. All these factors (design of SPATT filter, nature of the adsorbent substrate, quantity of resin, time of deployment, environmental factors) have probably an influence on the adsorption of toxins into SPATT devices, so that more studies are needed to lead to a standardization of the methodology that would be useful to allow inter-laboratory comparisons. However, the use of SPATT technology alone would not be acceptable for regulatory monitoring of shellfish and fish toxicity since it does not conform to AOAC guidelines [45]. As is the case for phytoplankton observations, SPATT technology may be useful as a supplementary technique, since it provides indications on the potential presence of biotoxins in the environment; however, it does not account for all eventualities relevant to food safety, e.g., the non-detection of biotransformed toxins naturally occurring in foodstuffs or the fact that SPATT cannot mimic the elimination of toxins by aquatic organisms. Analyses of the toxic metabolites really present in shellfish or fish species thus remain necessary to ensure their edibility.

Before passive sampling is approved for regulatory purposes to reinforce monitoring of HABs and associated aquatic toxins in ongoing risk assessment and public health management programs, it could potentially be useful as an alert tool. Indeed, SPATT technology has been successfully used in some studies as an early warning system to detect biotoxins prior to the occurrence of a bloom event and subsequent contamination of shellfish. For example, during the initial study that has introduced SPATT technology, increase in DSP toxins was observed in SPATT bags several weeks before shellfish toxicity reached its peak [21]. Likewise, a next study showed that SPATT devices successfully signaled the presence of DA in seawater three and seven weeks prior to *Pseudo-nitzschia* bloom onset, and seven and eight weeks prior to the detection of DA in sentinel shellfish samples [45]. However, in contrast, other studies obtained results that were not in favor of the potential use of SPATT technology as an early warning system. For example, SPATT devices did not enable the forecasting of shellfish contamination on the west coast of Ireland as the increase in diarrheic toxin concentration occurred at the same time in mussels and in SPATT bags [35]. Similarly, in another study, the detection of *Dinophysis* cells took place several weeks before toxins were actually detected in SPATT devices [40]. Thus, further long-term monitoring studies examining HABs prevalence and toxicity, as well as toxin accumulation in shellfish and fish, in parallel to SPATT field experiments, are needed to firmly validate the use of SPATT devices as an early warning tool of foodstuffs toxicity events. Nevertheless, when coupled with other current monitoring techniques such as microalgae/cyanobacteria cell monitoring and foodstuff testing, SPATT passive sampling could advantageously contribute to the ongoing reinforcement of human health risk assessment and management programs worldwide. For example, MCs have often been detected using SPATT when simultaneous discrete water samples have failed to detect them in a given waterway or waterbody, making SPATT a more sensitive indicator of the prevalence of toxins than traditional discrete samples [41,107]. Furthermore, this approach also shows great potential as a tool for fundamental research into the chemical ecology (i.e., toxins and other secondary metabolites) of aquatic microorganisms.

## Figures and Tables

**Table 1:** Lipophilic toxins successfully detected with SPATT technology, using different adsorbent substrates.

Toxins Detected	Adsorbent Resins Tested	References
**DSP toxins and PTXs** -OA, OA-D8 -DTX1, DTX2 -PTX2, PTX2sa, 7-epi-PTX2sa, PTX12, a PTX1 isomer, a PTX3 isomer, a PTX-11 isomer	-Diaion^®^ HP20, HP2MG -Sepabeads^®^ SP207, SP700, SP850, SP825L -Amberlite^®^ XAD4, XAD761 -Dowex^®^ Optipore^®^ L-493 -Oasis^®^ HLB -Strata-X^®^	[21,23,33,34,35,36,37,40,42,43,48,49,50,51,52,53,54,55,56,57,58]
**YTXs** YTX, homoYTX	-Diaion^®^ HP20, HP2MG -Sepabeads^®^ SP207	[21,35,48,49,57]
**AZP toxins** AZA1, AZA2, AZA3, AZA-59	-Diaion^®^ HP20 -Sepabeads^®^ SP700 -Oasis^®^ HLB -Strata-X^®^	[33,34,35,36,48,58,59]
**Cyclic imines** -13-desmethyl-SPX-C, 13,19-didesmethyl-SPX-C, iso-SPX-C, 20-methyl-SPX-G- PnTX-E, PnTX-F, PnTX-G-GYM-A	-Diaion^®^ HP20 -Sepabeads^®^ SP700 -Amberlite^®^ XAD761 -Oasis^®^ HLB -Strata-X^®^	[23,31,34,35,36,39,44,48,53,57,58]
**Ciguatoxins** P-CTX-1B, P-CTX-3C, P-CTX-3B, *iso*-P-CTX-3B/C	Diaion^®^ HP20	[30,32]

**Table 2:** Hydrophilic toxins successfully detected with SPATT technology, using different adsorbent substrates.

Toxins Detected	Adsorbent Resins Tested	References
**ASP toxin** DA	-Diaion^®^ HP20 -Sepabeads^®^ SP207, SP207SS, SP700	[45,48,104,105]
**PSP toxins** -STX, neo-STX -GTX1, GTX2, GTX3, GTX4 -C1, C2	-Diaion^®^ HP20 -Sepabeads^®^ SP700 -Computationally designed polymer	[45,106]
**MCs and NODs** MC-LR, MC-YR, MC-RR, MC-LA, MC-LF, MC-Desmethyl-LR, [Dha7] MC-LR, NOD	-Diaion^®^ HP20 -Sepabeads^®^ SP700	[38,41,46,105,107,108,109]
**ANTX**	-(Norit^®^ GAC -830, -1020, -1240) -Darco^®^ PAC G-60 -(Carbograph) -WCX -Strata –X^®^, -X-C, -X-CW -Oasis^®^ MCX -AG^®^ 50W-X4, (501-X8) -(Diaion^®^ HP20) -Amberlite^®^ IRP-64	[47,110]
**CFP toxins** MTX-1, putative MTX-3	Diaion^®^ HP20	[30,32]
Into brackets: resins that were not effective for the targeted toxins		
